# Chemical Composition, Antimicrobial and Antioxidant Activities of the Volatile Oil of *Ganoderma pfeifferi* Bres

**DOI:** 10.3390/medicines3020010

**Published:** 2016-04-28

**Authors:** Mohamed Al-Fatimi, Martina Wurster, Ulrike Lindequist

**Affiliations:** 1 Department of Pharmacognosy, Faculty of Pharmacy, Aden University, P.O. Box 5411 (Maalla) Aden, Yemen; 2 Department of Pharmaceutical Biology, Ernst-Moritz-Arndt-University Greifswald, D-17487 Greifswald, Germany; wurster@uni-greifswald.de (M.W.), lindequi@uni-greifswald.de (U.L.)

**Keywords:** *Ganoderma pfeifferi*, GC-MS, volatile oil composition, antimicrobial, antioxidant

## Abstract

In a first study of the volatile oil of the mushroom basidiomycete *Ganoderma pfeifferi* Bres., the chemical composition and antimicrobial and antioxidant activities of the oil were investigated. The volatile oil was obtained from the fresh fruiting bodies of *Ganoderma pfeifferi* Bres. By hydrodistillation extraction and analyzed by GC-MS. The antimicrobial activity of the oil was evaluated against five bacteria strains and two types of fungi strains, using disc diffusion and broth microdilution methods. In addition, the antioxidant activity of the oil was determined using DPPH assay. Four volatile compounds representing 90.5% of the total oil were identified. The majority of the essential oil was dominated by 1-octen-3-ol (amyl vinyl carbinol) **1** (73.6%) followed by 1-octen-3-ol acetate **2** (12.4%), phenylacetaldehyde **3** (3.0%) and 6-camphenol **4** (1.5%). The results showed that the Gram-positive bacteria species are more sensitive to the essential oil than Gram-negative bacteria. The oil showed strong antimicrobial activity against *Staphylococcus aureus* as well as *Candida albicans*. Moreover, the oil exhibited strong radical scavenging activity in the DPPH assay. This first report on the chemical composition and biological properties of *G. pfeifferi* volatile oil makes its pharmaceutical uses rational and provides a basis in the biological and phytochemical investigations of the volatile oils of Ganodermataceae species.

## 1. Introduction

The genus *Ganoderma* P. Karst. belongs to the family Ganodermataceae, class *Basidiomycetes.* It includes more than 200 species widespread all around the world. Mainly in East Asia, some species are used in traditional medicine and as a dietary supplement to prevent and treat cancer, hypertension, diabetes, skin ulcerations and many other diseases [1]. In contrast to *Ganoderma lucidum*, the most studied and used species of the genus *Ganoderma* [2,3], *Ganoderma pfeifferi* Bres., is less investigated. The occurrence of this parasitic basidiomycete is limited to Europe. It is living on *Fagus*, *Aesculus*, *Acer*, *Fraxinus*, and *Quercus* species [4]. Previous studies reported that the extracts and some isolated compounds from *G. pfeifferi* have antimicrobial, cytotoxic, anticancer and antiviral activities [5,6,7,8]. The antimicrobial activities of the dichloromethane extract are mainly caused by the meroterpenoids ganomycin A and B [5]. The antimicrobial activity of the ethanol extract is caused by the phenolic derivatives 2,4,5-trihydroxybenzaldehyde and 2,5-hydroxybenzoic acid [6]. The purpose of this study is to analyze the chemical composition and some biological activities of the volatile oil of *G. pfeifferi.* There are only few reports about the volatile components of basidiomycetes such as *Boletus edulis*, *Pleurotus ostreatus* [9], *Inonotus obliquus* [10], *Laetiporus sulphureus* [11], *Lentinus boryanus*, *L. edodes* [12], *Polyporus sulfurous* [13], *Trametes gibbosa* [14] and *Tricholoma matsutake* [15]. Ziegenbein *et al.* [16] identified 65 compounds in the essential oil from fruit bodies of *Ganoderma* *lucidum*. Major ones were trans-anethole (9.1%), *R*-(−)-linalool (4.4%), *S*-(+)-carvone (4.4%), 2-pentylfuran (2.8%), α-terpineol (2.7%) and *n*-nonanal (2.3%). The main components of the oil of the mycelium of *G. japonicum* were (*E*)-nerolidol (17.6%), (2*E*,4*E*)-decadienal (6.2%) and linalool (4.5%) [17]. To the best of our knowledge, no literature information is available on the chemical composition and biological activity of the volatile oil obtained from *G. pfeifferi*. 

## 2. Materials and Methods

### 2.1. Fungus (Mushroom) Material

The fresh mature fruiting bodies of *Ganoderma pfeifferi* were collected from the wild stump of *Fagus* sp. in Ludwigsburg nearby Greifswald, Northeast Germany, in August 2010. The collected basidiomycete was taxonomically identified by H. Kreisel at the Department of Microbiology, Ernst-Moritz-Arndt-University, Greifswald, Germany. A voucher specimen (FGG 001) was deposited at the Department of Microbiology, Ernst-Moritz-Arndt-University, Greifswald, Germany.

### 2.2.Volatile Oil Extraction

The *G. pfeifferi* fresh fruiting bodies were cut fresh in small pieces. The crushed fresh fruiting bodies (20 g) were hydrodistilled for 3 h in a Clevenger-type apparatus according to the European Pharmacopoeia. *n*-Hexane (1.5 mL) was used as a collector solvent. The oil yield was calculated on a dry weight basis by moisture free. The obtained volatile oil (VO) was subsequently dried over anhydrous Na_2_SO_4_ and stored at 4 °C before analysis. The volatile oil was subjected to GC-MS analysis.

### 2.3. Gas Chromatographic-Mass Spectral Analysis

GC-MS analysis: Analytical GC-MS system consisting of an Agilent 6890 N gas chromatograph and a mass selective detector (Agilent^®^5973 Network MSD, Agilent Technologies, Santa Clara, CA, USA) was used. Injection was done with Agilent^®^7683 Series Injector (Split 1:40 at 250 °C, 2.0 µL; carrier gas: helium 1.1 mL/min (60 kPa) at 110 °C; pressure rise: 6 kPa/min). The MS operated in the electron impact mode with an ionization energy of 70 eV. The oven program started with 1 min at 70 °C, the oven temperature was increased at 3 °C/min to 220 °C. Full scan mass spectra were acquired from 35–350 *m*/*z* at a rate of 4.5 scans/s and with a 5.00 min solvent delay. Chromatography was performed using a 30 m DB-5 column (J & W Scientific, Folsom, CA, USA) with 0.25 mm i.d. and 0.25 µm film thickness. The detected compounds were identified by processing of the raw GC-MS data with ChemStation G1701CA software and comparing with NIST mass spectral database 2.0 d (National Institute of Standards and Technology, Gaithersburg, MD, USA) and from retention indices and mass spectra of standard compounds. Relative amounts of detected compounds were calculated based on the peak areas of the total ion chromatograms (TIC).

### 2.4. Determination of Antimicrobial Activities

#### 2.4.1. Microorganisms

The following bacterial strains were employed in the screening: *Staphylococcus aureus* (ATCC 29213), *Bacillus subtilis* (ATCC 6059), *Escherichia coli* (ATCC 25922), *Pseudomonas aeruginosa* (ATCC 27853) and *Micrococcus flavus* (SBUG 16). As fungal strains, *Candida maltose* (SBUG 17) and *Candida albicans* (ATCC 90028) were used.

#### 2.4.2. Antimicrobial Assays

##### Agar Diffusion Method

Modified agar diffusion method [18] was used to determine antibacterial and antifungal activities. The bacterial cell suspension was prepared from a 24 h culture and adjusted to an inoculation of 1 × 10^6^ colony forming units per ml. Sterile nutrient agar (Immunpräparate, Berlin, Germany, D, 26 g agar/L distilled water) was inoculated with bacterial cells (200 μL of bacterial cell suspension in 20 mL medium) and poured into dishes to give a solid medium. Yeasts (1 × 10^5^ colony-forming units per mL) were inoculated into sterile Mueller-Hinton-agar (Becton Dickinson, Heidelberg, Germany) according to DIN E 58940–3 (DIN 2004) for the agar disc-diffusion assay. The volatile oil (10 μL for bacteria and 20 μL for the fungus) was applied on sterile paper discs (6 mm diameter, Schleicher & Schuell, GmbH, Dassel, D, Germany ref. no. 321860). The discs were deposited on the surface of inoculated agar plates. Plates with bacteria were incubated for 24 h at 37 °C and plates with yeasts for 48 h at 36 °C. Inhibition zone diameters around each of the discs (diameter of inhibition zone plus diameter of the disc) were measured and recorded at the end of the incubation time. An average zone of inhibition was calculated for the three replicates. Ampicillin, gentamicin and nystatin were used as positive controls.

##### Broth Micro-Dilution Assay for Minimum Inhibitory Concentrations (MIC)

The broth micro-dilution method described by Mann and Markham [19] with modifications was used to determine the MIC of investigated volatile oil against the above-tested bacterial strains. With sterile round-bottom 96-well plates, duplicate two-fold serial dilutions of oil (100 μL/well) were prepared in the appropriate broth containing 5% (*v*/*v*) DMSO. Two-fold dilutions of ampicillin, gentamicin and nystatin were used as positive controls. A bacterial cell suspension (prepared in the appropriate broth) of 100 μL, corresponding to 1 × 10^6^ CFU/mL, was added in all wells except those in columns 10, 11 and 12, which served as saline, volatile oil and media sterility controls, respectively. Controls for bacterial growth without volatile oil were also included on each plate. The final concentration of bacteria in the assay was 5 × 10^5^ CFU/mL. Plates were then incubated at 37 °C for 18 h overnight. After incubation, the MIC of each volatile oil was determined as the lowest concentration at which no growth was observed in the duplicate wells. Twenty microliters of a *p*-iodonitrotetrazolium violet solution (0.04%, *w*/*v*) (Sigma, Aldrich GmbH, Munich, Germany) was then added to each well. The plates were incubated for a further 30 min, and estimated visually for any change in color from yellow to pink indicating reduction of the dye due to bacterial growth. The highest dilution (lowest concentration) that remained yellow corresponded to the MIC. Experiments were performed in duplicate.

### 2.5. Determination of Radical Scavenging Activity

The free radical scavenging activity was measured by using 1,1-diphenyl-2-picryl-hydrazyl (DPPH) assay. This assay measures the free radical scavenging capacity of the investigated oils/extracts. Qualitative determination was done as described in Sievers [20]. Quantitative estimation was carried out according to the method of Brand [21]. The reaction mixture (total volume 1 mL) contained 500 μL of test volatile oil and 125 μL of DPPH in ethanol. Different concentrations of test volatile oil (10, 50, 100, 500 and 1000 μg/mL) were prepared while the concentration of DPPH was 1 mM in the reaction mixture. These reaction mixtures were taken into Eppendorf tubes and incubated at 37 °C for 30 min, the absorbance was measured at 517 nm. Percent radical scavenging activity by sample treatment was determined by comparison with the ethanol-treated control group. Ascorbic acid was used as the positive control. The DPPH radical concentration was calculated using the following equation:

Radical scavenging activity (%) = Absorbance control − Absorbance sample/Absorbance control × 100.

## 3. Results

### 3.1. Chemical Composition of the Volatile Oil

**Figure 1:**
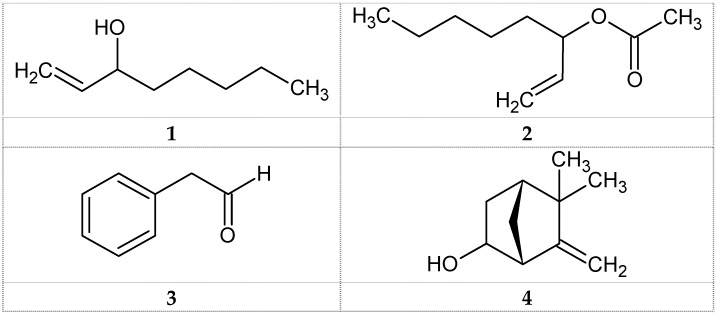
Structures of volatile compounds identified from *G. pfeifferi* using GC-MS analysis. **1**: 1-Octen-3-ol; **2**: 1-Octen-3-ol acetate; **3**: Phenylacetaldehyde; **4**: 6-Camphenol.

Hydrodistillation of the fresh fruiting bodies of the basidiomycete *G. pfeifferi* offered volatile oil (VO) in a yield of 0.25% on the fresh weight base. The obtained oil had a yellow color and a mushroom-like, fatty and floral odor. The composition of the VO is presented in Table 1, where all compounds are listed according to their elution from a DB-5 column. The GC chromatogram of the oil shows the presence of eight volatile compounds (Table 1); four were identified in the fresh oil of *G. pfeifferi*, accounting for 90.5% of the total compounds. The majority of the oil was dominated by the aliphatic unsaturated alcohol 1-octen-3-ol (amyl vinyl carbinol, 73.6%) **1** and its derivative 1-octen-3-ol acetate (amyl vinyl carbinol acetate, 12.4%) **2**, followed by the aromatic aldehyde phenylacetaldehyde (hyacinthin 3.0%) **3** and the oxygenated monoterpene 6-camphenol (1.5%) **4** (Table 1 and Figure 1). A previous study divided the main dominant volatile compounds of some mushroom species in three groups: C8 aliphatic compounds such as1-octen-3-ol, terpenes and aldehyde compounds [22]. Our results support this classification and show that the *G. pfeifferi* VO contained all three compounds classes whereby 1-octen-3-ol was the dominant volatile compound. Aliphatic C8 compounds seem to be the dominant class of compounds found in the oils of other mushrooms. 1-Octen-3-ol was previously identified as a dominant volatile compound, e.g., in the VO of the mushrooms *Agaricus bisporus* [23,24], *Boletopsis leucomelas* [25], *Pleurotus ostreatus* [26], *Pleurotus eryngii* var. *tuoliensis*, *P. cystidiosus* [27], *P. salmoneostramineus, P. sajor-caju* [28], *Spongiporus leucomallellus* [16] and *Trametes gibbosa* [14]. It was found also in the truffle *Hymenogaster luteus* var. *luteus* [29]*.* Its ester has not been reported in mushroom VO before. Phenyacetaldehyde has been identified in the VO of the mushroom species *Boletopsis leucomelas* [25] and *Pleurotus eryngii* var. *ferulae* [30]. It can be assumed that the unique flavor of the fruit bodies of *G. pfeifferi* is determined by the identified C8 compounds: 1-octen-3-ol (earthy, mushroom-like), its ester (fatty), phenylacetaldehyde (sweet aromatic, floral) and the monoterpene 6-camphenol (sweet scent).

### 3.2. Antimicrobial Activities of the Volatile Oil

The volatile oil from *G. pfeifferi* was assayed for its *in vitro* antimicrobial activity, using the agar diffusion and microdilution methods, towards five different bacterial species, representing both Gram-positive and Gram-negative bacteria, and two fungi strains (Table 2). VO expressed the highest antibacterial activity against Gram-positive bacteria such as *Staphylococcus aureus* (30 mm, MIC 0.3 mg/mL) and *Bacillus subtilis* (20 mm, MIC 0.6 mg/mL). *Escherichia coli* (15 mm, MIC 1.2 mg/mL) was the most affected Gram-negative bacterial strain. Furthermore, VO was significantly effective against *Candida albicans* (MIC 0.6 mg/mL) (Table 2). Few mushroom VOs have been tested for their antimicrobial activities such as from *Laetiporus sulphureus* [11], and *Pleurotus ostreatus* [26]. According to [26], the antibacterial activity of the VO of *P. ostreatus* depends greatly on the presence of 1-octen-3-ol. The compound is reported as the main constituent of the essential oils of some plants (7%–32%) which possess antibacterial or antifungal activities [31,32,33,34,35,36,37,38]. Phenylacetaldehyde is also known as an essential oil component with antimicrobial activity [31]. Probably, all components of the VO act synergistically. While the antimicrobial activities of different extracts of *G. pfeifferi* have been demonstrated in our previous studies [5,6], we present here the first report about the antimicrobial properties of the VO of *G. pfeifferi*.

### 3.3. Antioxidant Activity of the Volatile Oil

The volatile oil has been tested for 1,1-diphenyl-2-picryl-hydrazyl (DPPH) radical scavenging activity. It showed strong antioxidant activity with 90.9% inhibition at 1 mg/mL in comparison with ascorbic acid (96.2% at 1 mg/mL) (Table 3). Zhang *et al.* [39] attributed the antioxidant activity of the mushroom *Pleurotus ostreatus* to its high content of 1-octen-3-ol which constitutes about 73.6% of the oil of *G*. *pfeifferi*. The oxygenated monoterpene 6-camphenol and the aromatic aldehyde phenylacetaldehyde could also be taken into account for the antioxidant activity of *G. pfeifferi* VO.

## 4. Conclusions

To the best of our knowledge, we herein present the first report on the chemical composition and antimicrobial and radical scavenging properties of *G. pfeifferi* VO. The biological activities of the *G. pfeifferi* VO and extracts make their pharmaceutical uses rational and provide a basis for the future work with volatile oils of species of Ganodermataceae.

## Figures and Tables

**Table 1:** Chemical composition of volatile oil of fresh fruiting bodies of *G. pfeifferi*.

No.	RI	Compounds ^a^	Content %
1	973	unidentified	0.8
2	976	1-Octen-3-ol (Amyl vinyl carbinol, **1**)	73.6
3	980	unidentified	2.2
4	988	unidentified	2.2
5	1027	Phenylacetaldehyde (Hyacinthin, **3**)	3.0
6	1044	unidentified	4.3
7	1081	1-Octen-3-ol, acetate (Amyl vinyl carbinol acetate, **2**)	12.4
8	1447	6-Camphenol, (**4**)	1.5
		Total identified	90.5

^a^ Compounds listed in order of their elution on the DB-5 column; RI: Retention indices on the DB-5 column relative to C10-C20 *n*-alkanes.

**Table 2:** Antimicrobial activity of the volatile oil of *Ganoderma pfeifferi*.

Microbial Strains	Volatile Oil		Reference Antibiotics IZ (mm)		
	IZ (mm) 10 μL/disc	MIC (mg/mL)	Ampicillin 10 μg/disc	Gentamicin 10 μg/disc	Nystatin 100 μg/disc
**Gram-Positive Bacteria**					
*B. subtilis*	20	0.6	29	n.t.	n.t.
*M. flavus*	10	4.5	31	n.t.	n.t.
*S. aureus*	30	0.3	28	23	n.t.
**Gram-Negative Bacteria**					
*E. coli*	15	1.2	n.t.	15	n.t.
*P. aeruginosa*	8	n.t.	n.t.	18	n.t.
**Fungi Strains**					
*C. albicans*	20	0.6	n.t.	n.t.	25
*C. maltosa*	15	n.t.	n.t.	n.t.	25

**IZ:** Inhibition zone includes diameter of the disc (6 mm); n.t.: not tested.

**Table 3:** DPPH radical scavenging activity of volatile oil of *G.* *pfeifferi*.

Test Probe	Radical Scavenging Activity (%)				
Concentration	10 μg/mL	50 μg/mL	100 μg/mL	500 μg/mL	1000 μg/mL
Volatile oil	28.9	56.89	70.43	85.6	90.94
Ascorbic acid	48.5	89.5	95.8	96.1	96.2

## Abbreviations

The following abbreviations are used in this manuscript:

DPPH	1,1-diphenyl-2-picryl-hydrazyl (DPPH) assay
VO	Volatile oil
MIC	Minimal Inhibitory Concentration
